# Increased efficacy of regularly repeated cycles with OnabotulinumtoxinA in MOH patients beyond the first year of treatment

**DOI:** 10.1186/s10194-016-0634-9

**Published:** 2016-05-04

**Authors:** Simona Guerzoni, Lanfranco Pellesi, Carlo Baraldi, Luigi Alberto Pini

**Affiliations:** Headache and Drug Abuse Research Centre, Policlinico Hospital, University of Modena e Reggio Emilia, Via del Pozzo 71, 41100 Modena, Italy

## Abstract

**Background:**

Chronic migraine is one of the most common diseases in the world and it is often associated with medication overuse that can worsen the headache itself. Thus, it is important to adopt effective therapies to relieve pain and improve patients’ quality of life. The PREEMT studies have already demonstrated the effectiveness of OnabotulinumtoxinA in the treatment of chronic migraine. With this in mind, the aim of this real life observation has been to assess the clinical improvements as well as the impact on the quality of life of patients being regularly (every three months) administered this therapy.

**Methods:**

Data from 66 chronic-migraineurs treated with OnabotulinumtoxinA after failing previous therapies were collected. Only 57 of them were analysed since 9 discontinued the therapy due to administrative reasons. For every patient enrolled, headache frequency, analgesic consumption, pain severity, headache-related disability, health-related quality of life as well as anxiety and depression symptoms were collected through the Headache Index (HI), analgesic consumption rate in one day (AC), VAS score, Headache Impact Test (HIT-6) and the Short Form (36) Health Survey questionnaire Version 2 (SF-36®), Zung Self-Rating Anxiety Scale (ZUNG-A) and Zung Self-Rating Depression Scale (ZUNG-D), respectively.

All the changes vs baseline (Tx vs T0) were expressed as mean ± SD and analysed with a one-way ANOVA plus non-parametric Wilcoxon test, that was used for paired data for each subject.

**Results:**

As the number of injection increased, those patients injected regularly observed a statistically significant reduction in the headache frequency, pain intensity, headache disability score and an overall marked improvement in patients’ quality of life. There was also a significant reduction in anxiety and depressive symptoms as for the ZUNG-A and ZUNG-D scales scores. At any time point, those patients who stopped the therapy worsened their overall conditions as confirmed by quality of life parameters.

**Conclusions:**

This study outpoints that OnabotulinumtoxinA treatment is an effective treatment to reduce the headache-related disability and improve patients’ quality of life when patients are treated regularly every three months and consistently overtime. Therapy discontinuation leads to a general worsening of health-related quality of life. Long term treatment over one year confirms a consistently positive and sustained trend of improvement with a high safety profile.

## Background

Chronic migraine (CM) represents the most disabling condition among headaches, in particular when it is associated with drug abuse.

CM, that is defined as the presence of headache for 15 or more days per month in patients with migraine history, dramatically decreases quality of life (QoL) and determines an unquestionable morbidity in around 2 % of the general population [1, 2]. Treatment of CM is not worldwide scheduled and when analgesic overuse is present withdrawal is compulsory. Even if many chronic migraineurs need preventive treatments, there are well-done trials only for topiramate [3, 4]. After one month follow-up the majority of chronic-migraine patients respond to withdrawal, whereas the consistency is short and preventive drugs, alone or in combination, are ineffective.

OnabotulinumtoxinA (OnabotA) was approved by the FDA in 2010 for the preventive treatment of CM based on the results collected in the Phase III Research Evaluating Migraine Prophylaxis Therapy (PREEMPT) trial [5]. Globally were enrolled 1354 individuals and were evaluated the efficacy and safety of OnabotA as headache prophylaxis in chronic-migraineurs, treated every 12 weeks for up to one year [6]. Since then a few studies have recently demonstrated the effectiveness of OnatobA in patients with MOH [7, 8], but data regarding consistency of efficacy beyond one year of treatment are limited. Furthermore, after one year lack of response occurs in about one out of 9 patients and injections can be delayed, but not stopped, to four months in around 40 % of patients without relapsing in chronic headache [9].

Mechanism of action of OnabotA involved in reducing migraine attacks in CM has not been completely elucidated yet. However, it has been hypothesized that the release of neuropeptides induces vasodilation in meningeal areas and neurogenic inflammation so that repeated episodes of activation of the trigemino-vascular system can sensitize central pain pathways and lead to migraine chronicization [10]. OnabotA inhibits the neurotransmitters release and prevents neurogenic inflammation, inhibiting both peripheral [11] and central sensitization [12]. Moreover, it has been recently hypothesized that the antinociceptive effect could depend also by centrally mediated and axonal transport-dependent activity [13].

Patients with CM coming to our centre represent more than 80 % of Day Hospital (DH) and in-ward patients. The patients considered in this analysis have been treated only with OnabotA after almost one year of ineffective or slightly effective oral preventive treatments. Medication overuse headache (MOH) may complicate every type of headache and almost all the drugs employed for acute headache treatment are taken by MOH patients. These patients are difficult to treat due to both their refractoriness to anti-migraine prophylactic treatments and the frequent co-morbidities that often need a multidisciplinary approach which might lead to the prescription of a large amount of drugs.

In this observational study we included patients suffering from CM with MOH able to fill out diaries with no lack of information, and who referred to our Headache Clinic between May 2012 and May 2015. The treatment with OnabotA (Botox®, provided by Allergan) is an important therapeutic option both for its efficacy and for the safety profile [14]. The aim of our retrospective evaluation was to assess the efficacy, consistency and safety of OnabotA for repeated treatment cycles administered regularly every 3 months in a population with severe CM and MOH.

## Methods

In the Modena University Headache Centre we performed a retrospective study in a sample of 66 patients with a diagnosis of CM associated with medication overuse according to the classification ICHD-III (beta). The study was approved by the Ethical Provincial Committee of Modena (protocol n. 729/E.C., file 334/15) and conducted in compliance with the latest version of the Declaration of Helsinki.

### Aims of the study

The primary endpoint was to evaluate the reduction in the headache index (HI, number of headache days/days observed) and analgesic consumption (AC, number of analgesic doses/days observed) in patients treated consistently with OnabotA up to 7 cycles compared with the baseline.

Secondary endpoints were the assessment of several parameters mirroring the QoL by using self–reporting scales (SF-36 and HIT-6 scales), anxiety and depression (Zung A and D scales) and pain intensity (VAS scale) at the beginning and at every injection session.

### Outcome measures

Participants recorded headache characteristics using consecutive monthly headache diaries that were completed since 3 months before starting OnabotA and continued throughout the entire treatment period.

The headache frequency was calculated as the number of headache/migraine days over one month of observation and reported as Headache Index (HI). The analgesic consumption was calculated as the number of analgesic taken every month and reported as analgesic consumption (AC).

The intensity of pain was scored by a visual analog scale (VAS) with scores ranging from 0 = no pain to 10 = most severe pain, indicating the maximum pain score at every day and computing the mean value in the observed month.

Headache-related disability and health-related QoL were assessed at every injection session by using the six-item Headache Impact Test (HIT-6) (HIT-6 Scoring Interpretation Italy (Italian) Version 1.1 ©2001 QualityMetric, Inc. and GlaxoSmithKline Group of Companies) and the Short Form (36) Health Survey questionnaire Version 2 (SF-36®) (Istituto Ricerche Mario Negri, Milano, Italy). Each HIT-6 question was scored as never (6 points), rarely (8 points), sometimes (10 points), very often (11 points) or always (13 points) with questions 4–6 relating to the past 4 weeks, for a total score of 36–78 (60 = severe impact, 56–59 = substantial impact, 50–55 = some impact, and ≤49 = little to no impact).

The SF-36 contains eight scales relating to two summary measures (physical component and mental component summary scores). Each scale is scored between 0 = poor QoL and 100 = good QoL [15].

Anxiety and depression symptoms were assessed at every step of treatment with OnabotA according to the Zung Self-Rating Anxiety Scale (ZUNG-A) as well as Zung Self-Rating Depression Scale (ZUNG-D).

The Zung Self-Rating Depression Scale is a short self-administered survey which describes the depressed status of a patient. There are 20 items on the scale that deal with affective, psychological and somatic symptoms associated with depression. For every item there is a list of four answers that patient can chose and everyone of them is associated with a specific mark. The higher the overall mark, the worse the depression status. In a similar way, the Zung Self-Rating Anxiety Scale is a self-administered survey whose score represents the anxiety status in a patient. Also in this scale there are 20 items. A patient is asked to provide an answer to each item and every answer is associated with a mark. The total score is calculated based on the sum of the single ones and provides an overall idea of the anxiety status of the patient [16].

### Demographic data

We reviewed medical reports of 66 patients. However, in this report we computed the data of only 57 patients since they represent the ones who were injected regularly every 3 months without interruption until up to cycle 7 at least. In our real life experience, we have an additional number of patients that have received up to 13 repeated cycles. However, they are not considered in the current analysis and will be described in a future data-set.

All data were collected by the clinical database in use in our Centre.

The analysis was performed on patients referring to the Headache Study Centre of Modena University, between May 2012 and May 2015. All the patients fulfilled criteria for CM and MOH as for the ICHD-III (beta). Moreover, for this study we selected patients with chronic (daily or near daily) headache suffering since ≥1 year and taking ≥1 analgesic/day. According to this, these patients should be considered severe case of CM and MOH. This criteria was chosen to consider a consistent sample. Those patients who were not fulfilling these parameters were not included.

The demographic data are summarized in Table 1. These patients used a variety of antimigraine and pain killer drugs. The data of primary and secondary drugs overused at the start of Botox ® treatment are reported in Table 2.

**Table 1:** demographic characteristics

Sex	Number	Age	BMI (kg/m^2^)
Male	11	49 ± 11	25.3 ± 2.8
Female	46	54.2 ± 13	20.4 ± 2
All (Mean ± SD)	57	50.5 ± 13.7	22.8 ± 2.3

**Table 2:** Overused drugs

	Overused drugs		All
	Primary	Associated	
Triptans	35	7	42
NSAIDs	9	10	19
Codeine containing drugs	6	8	14
Mixtures (Difmetrè®)	5	4	9
Tramadol	2	0	2
All	57	29	86

Nine patients discontinued the treatment as a consequence of regional regulatory reasons due to a delay of the registration procedure in our Regional Health Care System. They stopped the treatment at different times. Then, they were called and interviewed by phone from 4 to 6 months after the discontinuation to follow up on their conditions. They were also asked if they wished restarting the therapy with OnabotA.

Their results and considerations are reported apart for discussion.

### Treatment

All the patients were treated with OnabotA according to the PREEMPT paradigm (155 U every three months into 31 injection sites) [5].

At day 0 (baseline), participants received OnabotA injections and afterwords every three months according with the PREEMPT protocol. Injections were administered by an expert clinician as for the fixed-dose paradigm across seven specific head-neck muscle areas.

### Safety

All patients were counted only once for each adverse event (AE) when multiple occurrences of the same AE were reported, except that in the by-treatment-cycle analysis each new onset of an AE was counted in the cycle in which it started. An on-going AE was not counted in subsequent cycles unless it worsened.

### Statistical analysis

All data were pooled and summarized with descriptive statistics and appropriate tests were performed. Continuous variables are reported as mean ± SD, categorical ones are reported as subjects-counts and percentages. The mean change from baseline (and the 95 % confidence interval for the mean change) for the number of headache days at the final treatment cycle was computed. The change of the outcome measures at each time point versus the baseline value was compared using a one-way ANOVA plus not parametric Wilcoxon test (Tx vs T0). Changes in the explored endpoints among patients who discontinued therapy were studied performing a paired *t*-test.

Data in the Tables and Figures are expressed as mean ± SD.

## Results

The enrolled patients were older than those already described in literature [17, 18]. This is probably due to the restrictive parameters of choice that selected patients with a long history of headache and with numerous attempts to discontinue analgesic use. In the Table 1 are reported also the number of treatment with Botox® every three months as reported in the schedule of protocol. The enrolled patients were 46 Female and 11 Male, aged between 19 and 77 years (mean 50.5 ± 13.7), with a normal BMI (mean 22.8 ± 2.3).

In the Table 2, we report the types of overused drugs. Many patients referred a constant and daily use of more than one type of drug, in some cases of different classes. Otherwise, there were patients using regularly two types of triptans or two types of NSAIDs (Not Steroidal Anti Inflammatory Drugs) simultaneously. We indicated as primary drug overused the drug used daily as baseline to prevent the incoming headache. Associated drugs were the drugs used in the case of persistent pain or in the case of recurrent headache and used always for more than 15 days/month.

Only one patient took triptans and a pill containing barbiturate (butalbital in association with phenazon and caffeine, reported in the table as mixture). 35 out of 57 patients (61 %) reported the overuse of a single drug. Overall, the most overused drugs were triptans, which were primarily overused by 42 patients.

**Fig. 1:**
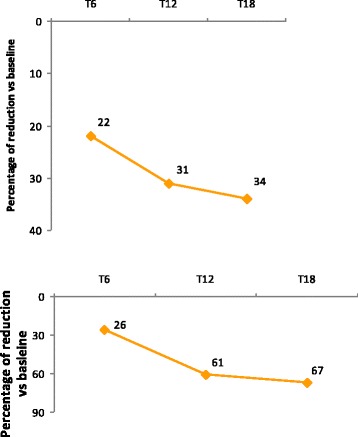
Change in percentage of HI (upper) and AC (lower) from T6 to T18 compared to baseline. Our observational results confirmed that already after 2 treatment cycles the HI was powerfully reduced of 22 % vs the baseline value and it kept consistently reducing overtime down to 34 % at T18 (at the cycle 7). This clinical improvement was further raised up by a simultaneous marked trend of reduction in AC vs the basal status that reached a 67 % at T18

The complete results of the primary endpoint are reported in the Table 3 as the number of days with headache for month (HI) and mean number of analgesic taken every day (AC). The results show a statistically significant reduction of the Headache Index and Analgesic Consumption at any injection session compared with the basal status (*P* < 0.001). To better explain the impact of the clinical response, in the Fig. 1 we have analyzed the results also in terms of the percentage of reduction of HI (in the upper graph) and AC (in the lower graph) from the sixth-month injection (T6) to the eighteen-month one (T18). They outline the high efficacy response in terms of reduction of headache days and acute drug intake achieved when patients are offered regularly repeated treatments. Our observational results support the importance of consistently treat patients to sustain and improve their clinical outcomes. Already after 2 treatment cycles the HI was powerfully reduced of 22 % vs the baseline value and it kept consistently reducing overtime down to an additional 12 % at T18 (at the cycle 7). Simultaneously, the AC greatly decreased with an even higher trend vs the basal status from 26 % at T6 to 67 % at T18.

**Table 3:** Headache Index (HI) and Analgesic daily Consumption (AC)

	T0 (*n* = 57)	T3 (*n* = 57)	T6 (*n* = 50)	T9 (*n* = 36)	T12 (*n* = 20)	T15 (*n* = 13)	T18 (*n* = 7)
Headache Index	0.98 ± 0.09	0.86 ± 0.24*	0.77 ± 0.3*	0.72 ± 0.34*	0.69 ± 0.29*	0.52 ± 0.29*	0.65 ± 0.36*
Analgesic consumption	1.79 ± 1.59	1.47 ± 1.67*	1.33 ± 1.90*	0.96 ± 0.97*	0.70 ± 0.43*	0.53 ± 0.30*	0.61 ± 0.42*

*P < 0.0001, one Way ANOVA + Wilcoxon test for paired data vs T0

**Fig. 2:**
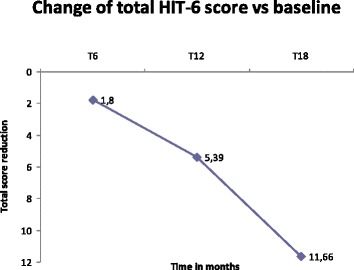
Change vs baseline in the HIT-6 score questionnaire from T6 to T18. Absolute HIT-6 Score reduction rate. The results show a positive and constant trend of reduction, and highlight the marked improvement in the QoL as demonstrated by a total HIT-6 score decrease of almost 12 points at T18 vs baseline (about 5 times higher the minimal important difference)

**Fig. 3:**
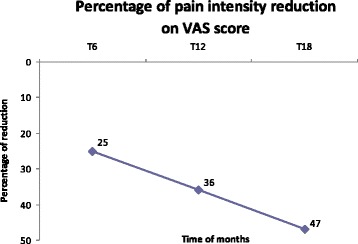
Change in percentage of VAS score vs baseline from T6 to T18. A positive and sustained trend of pain intensity reduction was observed overtime in our patients vs baseline

The secondary endpoints results are reported in the Table 4. We firstly evaluated the HIT-6 score, which was significantly lower from the fifth injection (*P* < 0.05 for T12, *P* < 0.01 for T15 and T18), while the VAS score was significantly lower already after the second injection (*P* < 0.01). To better explain headache-related disability as well as pain relief, we’ve reported the absolute HIT-6 score reduction rate and the percentage of VAS reduction in the Figs. 2 and 3, respectively. The results not only have showed a very positive and constant trend of reduction, but also highlight the valuable impact on the QoL of our patients by a total HIT-6 score decrease of almost 12 point at T18 vs baseline that is about 5 times higher the minimal important difference. Our patients observed a very impactful reduction in their disability further supported by a important and sustained pain reduction of up to 47 % at T18.

**Table 4:** Secondary endpoints

	T0 (*n* = 57)	T3 (*n* = 57)	T6 (*n* = 50)	T9 (*n* = 36)	T12 (*n* = 20)	T15 (*n* = 13)	T18 (*n* = 7)
HIT-6 score	63.94 ± 6.91	63.6 ± 6.89	62.14 ± 8.06	61.94 ± 8.13	58.55 ± 9.40*	55.7 ± 9.24**	52.28 ± 8.69**
VAS score	7.98 ± 1.26	6.98 ± 1.57**	6.01 ± 1.89**	5.19 ± 1.82**	5.14 ± 1.61**	4.69 ± 1.75**	4.25 ± 1.48**
SF-36 Mental	48.30 ± 21.68	50.41 ± 22.50	51.71 ± 22.35	52.82 ± 23.44	59.41 ± 21.16	67.8 ± 16.8**	73.90 ± 20.26*
SF-36 Physical	46.35 ± 18.90	46.85 ± 20.21	49.17 ± 19.90	48.94 ± 19.80	52.58 ± 24.69	62.11 ± 23.5*	70.18 ± 23.22*
ZUNG-A	41.27 ± 10.26	20.69 ± 9.82	40.76 ± 10.39	39.43 ± 9.74	37.48 ± 9.43	35.08 ± 7.11*	34 ± 6.11
ZUNG-D	42.95 ± 11.25	42.80 ± 11.31	43.4 ± 12.08	41.32 ± 10.03	38.23 ± 10.39	35.69 ± 7.82	37.14 ± 7.65

*P < 0.05; **P < =0.01 ANOVA followed by Wilcoxon test for paired data versus T0

The SF-36 Mental and Physical scores were significantly higher only after the sixth injection.

Data obtained with Zung-A and Zung-D scales were inconsistent and probably this tests aren’t able to outline the changes related to the headache modifications, when considering the modifications obtained in the QoL scores.

As described above, patients who discontinued because of regulatory reasons were considered separately on the same items, and not pooled together due to the differences in times of discontinuation and consistency of treatment.

Overall, the patients who discontinued the treatment suffered of a general worsening of their conditions within 3 months. This worsening was different in terms of outcomes according to the patient. However, a more generalized marked negative impact in the QoL was consistent for all of them as confirmed by the significative decrease in both the SF-36 scales. In one case, we also recorded that the AC newly increased up to 65 % when comparing the values between before the interruption and the first follow up visit to restart the OnabotA treatment.

### Adverse events

Overall, 33 of 57 patients (58 %) did not refer any AEs during the treatment period. No serious AEs were reported at any time. AEs considered definitely or probably related to the treatment occurred in 12 cases (20 %), and were considered mild or (two cases) moderate and were fully resolved after a short time. They included cervical muscle pain, injection site bruising, syncope following injection, tension type headache and sensation of weight on neck. There were no significant changes in any of vital signs from baseline and final time for each patient. These results confirm the OnabotA tolerability profile already seen in real-life studies [14].

## Discussion

CM represents migraine possible evolution from an episodic form, and MOH is the main and frequent complication or evolution of these patients. In 2012, Martelletti reviewed therapeutic agents for re-prophylaxis after detoxification in patients with CM with and without medication overuse and reported that only topiramate and local injection of OnabotA showed significant similar efficacy. Moreover, OnabotA was associated with a better adverse events profile [19]; those results have also been confirmed by Ahmed et al. in 2011 [7].

More recently, a review reported all the studies for CM-MOH treatment and among articles investigating a specific kind of preventive treatment, on a total of 17 studies, 5 of them reported the effect of OnabotA and 3 studies discussed the effect of topiramate, while a few others reported the effects of other drugs, such as valproic acid, nabilone and pregabalin [20].

In our experience, the primary endpoints rapidly reached a statistical significance compared with baseline already after three months, confirming the results recently outpointed by Negro et al. [14]. An even more clinically and marked relevance was obtained at the T6 session both for HI and AC. In particular, Figure 1 shows that within 3 months, between the third and the fifth injection there is a reduction of the HI of about 9 %. Moreover, between the fifth and the seventh injection there is a reduction of 3 % of the HI. Globally, between the third and the seventh injection the headache days within a month decreased of about 13 %. It is possible hypothesize that the high reduction in HI and AC in the first period reflects both the pharmacological and the placebo effect together that we can collect at the beginning of the treatment..In fact, also in the PREEMPT extension the gap between treated and placebo were significant in the double blind period, whereas after that both the curves were parallel [6].

In terms of secondary endpoints, the HIT-6 was significantly reduced only after 12 months and the score ameliorated in the subsequent sessions. Fig. 2 shows that there is a global reduction of 10 % in the HIT-6 Score between the third and the seventh injection. This result has also been recently confirmed by Khalil et al. [21].

Anxiety and depression scales showed a small improvement and only in a session we registered a significant reduction of anxiety scale. This result disagrees with Boudreau who reported improvements of the same parameters we used, out of depression. In fact, in a six months study they reported that Botox® was effective over headache frequency and analgesic consumption, headache impact on QoL, related disability was effective in reducing also depression and anxiety symptoms [22]. The discrepancies are probably due to the different tests used in measuring anxiety and depression symptoms, as well as the characteristics of sample. In fact, in our sample the scores of the specific tests for QoL at baseline were lower than in other series confirming the severity of our patients as compared with other series reported in literature [23].

The evaluation of QoL with SF-36 questionnaire showed a significant improvement after one year of treatment. Both mental and physical scores increased during all the observation period confirming a very positive trend. Furthermore, these scores worsened rapidly and with statistical significance after therapy discontinuation.

In our patients no serious AEs were recorded at any time. AEs treatment-related occurred in 20 % of cases, all of mild or moderate (two cases) intensity and were spontaneously and rapidly resolved within some hours and without specific treatments. All these AEs were of type 1 and no drop-out were related to these events.

## Conclusions

There are two main items to be discussed in this study:

- The consistency of efficacy and sustained benefit of the OnabotA treatment over the time
- the effect on analgesic drug use over the time in MOH patients

The consistency of Botox® in the long term treatment was not resolved in the PREEMPT studies, such as the questions about the overall duration of treatment, the length of withdrawal of treatment at scheduled times, and if these interruptions have to be done are still in debate.

This retrospective survey on a series of severe patients we followed for many years and treated with fixed doses suggests a number of points we can discuss.

The improvement of the primary endpoints was observed rapidly and consistently during all the observed treatment period. Those patients who discontinued the treatment because of legal reasons, worsened after some weeks and renewed improvement after 3 or 6 months after being administered a new OnabotA treatment.

Patients who continued without any interruption showed an increasing and progressive improvement without reduction of efficacy both in HI and in AC. The score related to the QoL improved only after one year of treatment thus suggesting that the real improvement and the exit from daily headache needs many months to be consolidated.

The second point we want to discuss concerns the role of the analgesic daily use (so called overuse or misuse or abuse). The patients we examined were all daily consumers of 1 to 5 analgesic drugs to reduce or try to prevent (unsuccessfully) their headache attacks.

There is a large debate on the role of analgesics and triptans in chronicization or sensitization of chronic migraine and headaches, but at the moment we would like to discuss only if the drug discontinuation was essential to obtain clinical improving. [24].

The results in literature indicate that OnabotA, without early discontinuation of the overused medication, was effective and well tolerated as headache prophylaxis in CM with MOH patients, even if these data were obtained only after post-hoc analysis [7, 17, 25].

Considering current available evidence, Chiang suggests discontinuation of the overused medication with the addition of preventive medication and hopes “Studies on preventive medications plus early discontinuation vs preventive treatment alone vs early discontinuation alone are needed” [20]. Our data shows that the discontinuation in drugs overuse, related to the decrease in AC score is linked with the reduction of VAS score and the headache-related disability according to the HIT-6 score change.

Our data can confirm this point based on our previous experiences according on the beneficial effect due to an early medication discontinuation even on the psychological structure and compliance of patients in the scheduled program [26, 27].

In conclusion, we can confirm that in accordance with literature data patients with chronic migraine CM and MOH are responsive to the use of OnabotA with an early discontinuation of medication overused. This treatment is also consistent and safe over long time. Furthermore, patients who discontinue the treatment are exposed at a high risk of a general health quality regression.

## Abbreviations

AC: analgesic consumption
AE: adverse event
ANOVA: analysis of variance
CM: chronic migraine
DH: day-hospital
HI: headache index
HIT: headache impact test
ICHD-III (beta): International Classification of Headache Disorders, Third Edition-BETA Version
MOH: medication overuse headache
NSAIDs: not steroidal anti inflammatory drugs
OnabotA: OnabotulinumtoxinA
PREEMPT: phase III research evaluating migraine prophylaxis therapy
QoL: quality of life
SD: standard deviation
SF-36: short form (36) health survey questionnaire version 2
VAS: visual analogic scale
ZUNG-A: Zung self-rating anxiety scale
ZUNG-D: Zung self-rating depression scale
